# Diverticular disease of the right colon

**DOI:** 10.1186/1756-0500-4-383

**Published:** 2011-10-06

**Authors:** Jasim M Radhi, Jennifer A Ramsay, Odette Boutross-Tadross

**Affiliations:** 1 Department of Pathology, McMaster University, Hamilton, Ontario, Canada

## Abstract

**Background:**

The incidence of colonic diverticular disease varies with national origin, cultural background and diet. The frequency of this disease increases with advancing age. Right-sided diverticular disease is uncommon and reported to occur in 1-2% of surgical specimens in European and American series. In contrast the disease is more prevalent and reported in 43-50% of specimens in Asian series. Various lines of evidence suggest this variation may represent hereditary differences. The aim of the study is to report all cases of right sided diverticular disease underwent surgical resection or identified during pathological examination of right hemicoloectomy specimens

**Methods:**

A retrospective review of all surgical specimens with right sided colonic diverticular disease selected from a larger database of all colonic diverticulosis and diverticulitis surgical specimen reported between January 1993 and December 2010 at the Pathology Department McMaster University Medical Centre Canada. The clinical and pathological features of these cases were reviewed

**Results:**

The review identified 15 cases of right colon diverticulosis. The clinical diagnoses of these cases were appendicitis, diverticulitis or adenocarcinoma. Eight cases of single congenital perforated diverticuli were identified and seven cases were incidental multiple acquired diverticuli found in specimen resected for right side colonic carcinomas/large adenomas. Laparotomy or laparoscopic assisted haemicolectomies were done for all cases. Pathological examination showed caecal wall thickening with inflammation associated with perforated diverticuli. Histology confirmed true solitary diverticuli that exhibited in two cases thick walled vessels in the submucosa and muscular layer indicating vascular malformation/angiodysplasia. Acquired diverticuli tend to be multiple and are mostly seen in specimens resected for neoplastic right colon diseases.

**Conclusion:**

Single true diverticular disease of the right colon is usually of congenital type and affects younger age group and may be associated with angiodysplasia in some cases. Multiple false diverticuli are more seen in association with caecal carcinoma or large adenomas. These are usually asymptomatic and are more seen in older patients. However this study dose not reflects the true incidence of the disease in the general population.

## Background

Diverticular disease of the colon becomes more common in well-developed countries. The prevalence depends on variable factors such as national origin, cultural background and consumed diet. The occurrence of this disease appears to increases with age [1]. Diverticula can develop anywhere in the large colon, however, they are most common in the left side. The pathophysiology of diverticular disease is rather complex and most likely related to the following factors, colonic motility disorders, changes in the colonic wall, chronic low-grade inflammation of the colonic wall, changes and imbalance of microflora and also visceral hypersensitivity [2]. Moreover, genetic factors may play a key role in the development of colonic diverticula. It is clearly established that there is strong association with decreased dietary fiber and lack of physical activity. In addition, some cases of diverticular diseases are found in patients with muscular diseases or abnormalities of the colon or complicate connective tissue diseases and neural abnormalities [3]. Human cancers produce several types of matrix metalloproteinase (MMPs) that are able to degrade extracellular matrix [4] and perhaps lead to the development of diverticular disease. Patients with this disease are usually asymptomatic; however, 10-20% of those affected may present with clinical symptoms, due to complication of diverticular diseases such as inflammation or hemorrhage. The incidence of diverticular disease is on the rise, due to the growing of the elderly population [4,5]

Caecal diverticular disease was first described by Potier in 1912 [6]. The incidence of diverticular disease of the caecum in North America remains low and probably representing approximately 1-2% of all cases of colonic diverticulosis. In the Orient, caecal diverticular disease is more common and may be as prevalent as those arising in the left side, representing 43 to 50% depending on the series [7]. It appears that caecal diverticulitis is becoming more recognized in Western literature, and is reported mostly in association with rather young age group population [8]. The most common clinical misdiagnosis of the right colon diverticular disease is acute appendicitis [9]. However, in retrospect most patients presented with dull pain which is typically of longer duration, and less likely to be associated with nausea and vomiting. Most reported cases in the literature highlighted the problematic preoperative diagnosis; however, it needs to be considered in patients with right lower abdominal pain [7,8]. During laprotomy the disease can closely mimic colonic adenocarcinoma [10]. The management in cases of a solitary diverticulum is either conservative or diverticulectomy if technically feasible. However, in complicated cases and those with caecal inflammatory masses, and when malignancy cannot be clinically excluded, right hemicolectomy is indicated. The surgical approach can be safely applied on those urgent cases with no major complications [11]. The aetiology of right sided colonic diverticula remains unclear, and the relation with angiodysplasia was rarely described in few cases [12].

## Methods

A retrospective review of McMaster Medical Center laboratory information system was conducted for all surgically resected colonic diverticular and diverticulitis cases for the period 1993-2010 to identify patients with right side diverticula. The study was approved and conducted according to Hamilton Health Science Research Ethic Board guidelines. The search revealed 465 patients underwent surgery for diverticular disease of the colon. There were 15 cases of right side colon diverticular diseases identified and selected for this study. We reviewed the patient's clinical information for age, gender, presentation, and type of surgery. The pathological specimens were retrieved and reviewed for the presence of single or multiple diverticuli, presence or absence of inflammation, perforation, angiodysplasia or malignancy. Diverticula consisting of a pouch of mucosa and muscularis mucosae projecting through and beyond muscular layer of the colon were labeled as false or pulsion diverticula and those with all layers of bowel wall were considered congenital or true diverticula.

## Results

Fifteen cases of caecal diverticula were identified. The clinical and pathological summary is presented in table 1. The patient's ages ranged from 23 to 87 years. These were consisting of 9 females and 6 males. Seven patients were admitted to hospital with a presumed clinical diagnosis of appendicitis. These cases presented with abdominal pain. Some patients complained of right lower quadrant pain and other with diffuse abdominal pain of few days duration. No nausea or vomiting and no urinary symptoms or history of inflammatory bowel diseases present. Two cases presented with bright red blood per rectum and one with a large enough bleed to require two units of packed red blood cells Three patients presented with acute abdomen with features of perforation and emergency exploratory surgery was performed. Preoperative abdominal CT scans were performed in a few cases in the present study. This showed segment of circumferential thickening of right colon with surrounding inflammatory changes. The finding suggested perforated neoplastic process or less likely diverticulitis. All cases of caecal diverticulitis underwent laparoscopic assisted right hemicolectomy resection, extended to open lapratomy in on case due to extensive adhesion. The other cases were incidental findings encountered during the examination of right hemicoloectomy specimens resected for colonic carcinoma or large villous adenoma.

**Table 1:** Highlighted the clinical and pathological features

Case	Age	Sex	Clinical Presentation	Pathology
1	23	M	Abdominal pain	Perforated caecal diverticulum with angiodysplasia
2	59	F	Caecal mass	Large villous adenoma incidental diverticulum.
3	80	F	Caecal ulcerated mass	Adenocarcinoma multiple diverticuli.
4	39	M	Abdominal pain	Perforated single diverticulum with angiodysplasia.
5	74	F	Abdominal pain and bleeding	Perforated single diverticulum with inflammatory mass.
6	25	M	Abdominal pain	Single inflamed diverticulum.
7	74	M	Caecal tumour	Adenocarcinoma incidental multiple diverticuli.
8	65	F	Caecal tumour	Adenocarcinoma incidental multiple diverticuli.
9	71	F	Caecal tumour	Adenocarcinoma incidental diverticulum.
10	68	M	Abdominal pain and bleeding	Single inflamed diverticulum with perforation.
11	55	M	Abdominal pain	Single inflamed diverticulum.
12	73	F	Abdominal pain	Single diverticulum Perforation.
13	29	F	Abdominal pain	Single diverticulum with diverticulitis.
14	59	F	Caecal tumour	Multiple small diverticula with large villous adenoma
15	71	F	Caecal tumour	Adenocarcinoma with multiple Diverticuli

**Figure 1:**
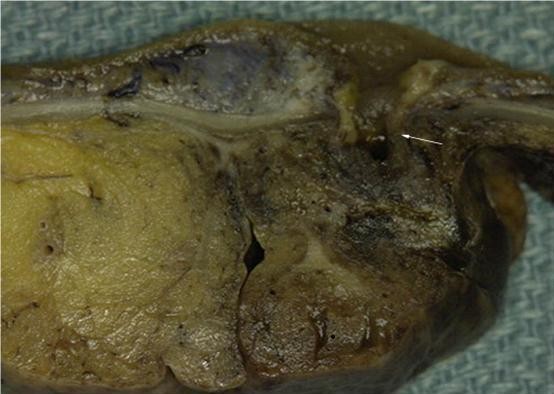
**Macro photograph of a perforated single true diverticulum of the caecum with surrounding inflammation**.

**Figure 2:**
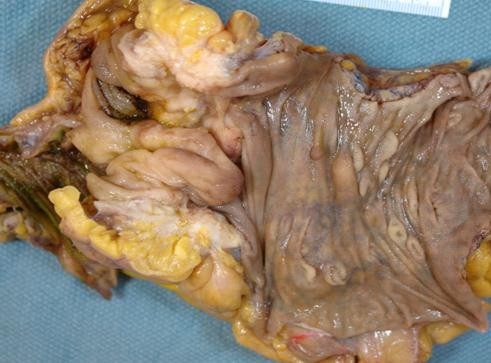
**Macro photograph of a false diverticulum (arrow) in right colon associated with ulcerated adenocarcinoma in the vicinity**.

**Figure 3:**
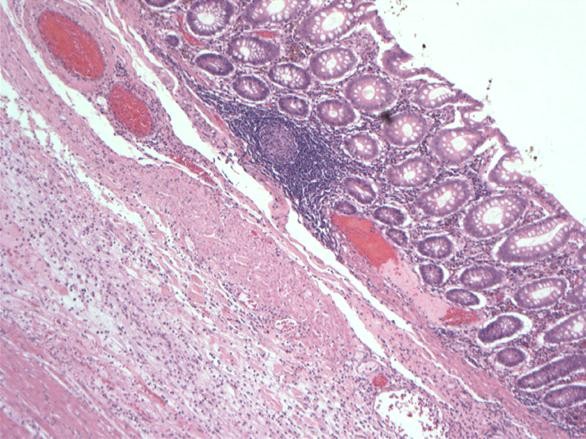
**This is a microphotograph of true diverticulum from an area with focal inflammation**.

**Figure 4:**
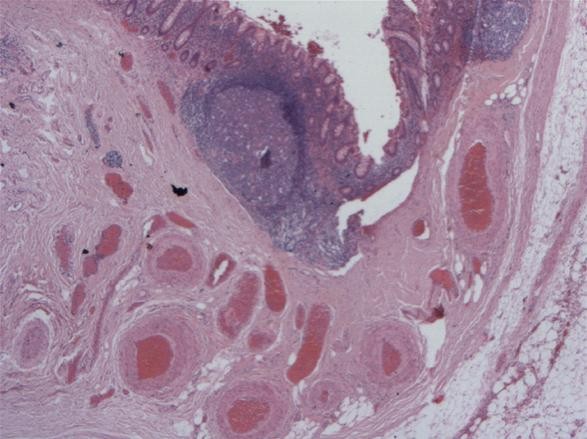
**Histology of angiodysplastic changes of submucosal vessels in the tip of a diverticulum**.

Pathological examination, showed single caecal diverticula with diverticulitis and perforation. There were inflammatory masses producing thickening of the caecal wall [Figure 1]. Caecal carcinoma was presented in five cases with multiple diverticula [Figure 2] and two cases with large villous adenomas. Histological examination confirmed true diverticula in four cases consisting of all layers of the bowel wall [Figure 3]. In addition, two cases demonstrated thick abnormal vessels with fibrointimal hyperplasia identified in the submucosa and muscularis propria [Figure 4]. The mucosa demonstrated thin-wall ectatic vessels. The overall features are compatible with angiodysplastic changes

**Figure 5:**
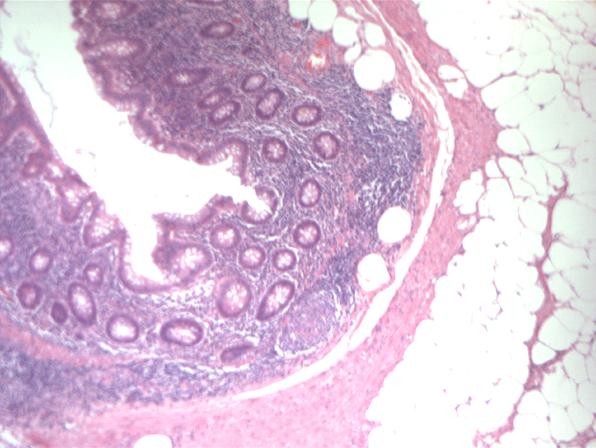
**Microphotograph of a false pulsion type diverticulum**.

The acquired diverticula were composed of herniated mucosa and submucosa with no evidence of inflammation [Figure 5]. There was no muscular hypertrophy of right colon in any of the cases. The appendix was either normal or adherent to the inflammatory masses in these cases.

## Discussion

Right sided diverticular colonic disease is rather uncommon in the Western World. The frequency of this disease is reported in approximately 1-2% of surgical specimens in European and American series, but may reach as high as 43-50% in Asian series [7]. Controversy persists concerning the origins of caecal diverticula. Right-sided diverticula occur more often in younger patients than do left-sided diverticula [1]. The majority of colonic diverticula are acquired in nature. These typically are characterized by herniation of mucosa and muscularis mucosa through the colonic wall. They usually are invested with a thin layer of submucosa that is forced out through the weak points in the muscularis propria and the tips ending in the colonic subserosa [1,3]. The weak points in the muscle coat represent the sites of entry of the nutrient vessels of the colonic mucosa. Diverticula are generally associated with increased colonic intraluminal pressure. The pathology showed thickened muscularis propria with normal or inflamed colonic mucosa. Caecal diverticula lack the muscular hypertrophy. Recent studies documented that aberrant activity of the matrix metalloproteinases play a role in changing the ratio of type1 to type 2 collagen in cases of diverticulitis, and also human cancer can produce MMPs that are leading to digestion of the extracellular matrix [4] which may play a significant role in the development of diverticular disease. The solitary caecal diverticula are usually congenital in origin, and thought arising as a sacular projection during the sixth week of embryonic development [10]. Rare instances of angiodysplasia are reported in association with true caecal and Meckel's diverticula [13].

Our findings support this association in cases of true diverticula. This correlation implicates a common aetiological factor. The presence of thick abnormal vessels claimed to trigger local haemodynamic/pressure changes render the background for diverticular disease. One study claimed the morphology of myenteric plexuses, and the ganglion cells differ significantly among segments of the human large intestine. Large intestines with diverticula had significantly more plexuses but significantly fewer ganglion cells than large intestines without diverticula [14]. The area of the nuclei of ganglion cells was also significantly smaller in large intestines with diverticula. This finding is difficult to investigate due to the presence of severe inflammation in true diverticula of the caecum...

Most patients with right sided diverticula are asymptomatic; however, patients may present with complications of diverticulosis. These include bleeding, diverticulitis, peridiverticular abscess, perforation with fistula formation [15]. Patients with true caecal diverticla are generally in the younger age group. Those subjects will present with right lower quadrant pain and are often thought to suffer from acute appendicitis. The diagnosis of right sided diverticulitis may subsequently be made in the operating room. It is difficult to differentiate caecal diverticulitis from acute appendicitis. More than 70% of patients with caecal diverticulitis were operated on with a preoperative diagnosis of acute appendicitis [10,11]. The correct preoperative diagnosis can be facilitated by ultrasound and computer tomography (CT), which are both highly cost-effective.

CT scanning is a sensitive means by which to detect caecal diverticulitis. The radiographic appearance of the disease; however, mimics that of appendicitis, unless more specific findings such as caecal diverticula or intramural abscess with adjacent inflammation are detected. CT and barium studies are complementary methods of examination that improve our ability to diagnose caecal diverticulitis and its complications [16-18].

MRI of right-side diverticulitis may reveal an out pouching of the right colon with associated circumferential wall thickening of the colon and surrounding inflammatory changes [19] Some authors suggested ultrasound and CT scan as routine use for abdominal pain of the right lower quadrant, which would probably reduce surgeries and hospital stays [4]. Recognition of specific imaging findings enables the radiologist to make the correct diagnosis and helps in establishing the appropriate surgical or medical therapy, thus avoiding unnecessary exploration or surgery for some of these surgical conditions, which mimicked acute appendicitis. When the diagnosis is made intraoperatively, the surgical management of the disease is controversial. Conservative management with antibiotics has been suggested for the uncomplicated caecal diverticulitis diagnosed intraoperatively. Excisional treatment for caecal diverticulitis prevents the recurrence of symptoms [20].

If technically feasible, aggressive resection with immediate right hemicolectomy should be considered in cases of extensive inflammatory changes, multiple diverticula and caecal phlegmon, or when neoplastic disease can not be excluded. This surgery can be safely performed even in the unprepared colon with few complications [21-23].

## Conclusion

In Western countries, diverticulosis mostly affects the left colon and the incidence of right sided diverticulitis is estimated around 2% of colonic diverticular disease. However, in Asia and countries with high Asian population, diverticular disease of the caecum and the ascending colon is more common than the left sided form of this disease. In Canada, despite the multicultural and ethnic mix, the incidence of this disease presented to surgery is rather low. Single diverticula are usually present in young patients and are prone to complication. Multiple diverticula are asymptomatic, finding in older patients mostly seen in association with carcinoma.

## Competing interests

The authors declare that they have no competing interests.

## Authors' contributions

JR contributed to the collection of clinical data, literature review and drafting of the manuscript. JAR and OBT contributed cases and reviewed the manuscript. All the authors gave final approval of the submitted manuscript.
